# Multiple-Junction-Based Traffic-Aware Routing Protocol Using ACO Algorithm in Urban Vehicular Networks

**DOI:** 10.3390/s24092913

**Published:** 2024-05-02

**Authors:** Seung-Won Lee, Kyung-Soo Heo, Min-A Kim, Do-Kyoung Kim, Hoon Choi

**Affiliations:** 1 Department of C4I Research, LIG Nex1, Seongnam 13488, Republic of Korea; kyungsoo.heo@lignex1.com (K.-S.H.); mina.kim@lignex1.com (M.-A.K.); kimdokyoung@lignex1.com (D.-K.K.); 2 Department of Computer Science and Engineering, Chungnam National University, Daejeon 34134, Republic of Korea; hc@cnu.ac.kr

**Keywords:** VANET, VANET routing protocol, traffic-aware, ACO algorithm, multiple junction

## Abstract

The burgeoning interest in intelligent transportation systems (ITS) and the widespread adoption of in-vehicle amenities like infotainment have spurred a heightened fascination with vehicular ad-hoc networks (VANETs). Multi-hop routing protocols are pivotal in actualizing these in-vehicle services, such as infotainment, wirelessly. This study presents a novel protocol called multiple junction-based traffic-aware routing (MJTAR) for VANET vehicles operating in urban environments. MJTAR represents an advancement over the improved greedy traffic-aware routing (GyTAR) protocol. MJTAR introduces a distributed mechanism capable of recognizing vehicle traffic and computing curve metric distances based on two-hop junctions. Additionally, it employs a technique to dynamically select the most optimal multiple junctions between source and destination using the ant colony optimization (ACO) algorithm. We implemented the proposed protocol using the network simulator 3 (NS-3) and simulation of urban mobility (SUMO) simulators and conducted performance evaluations by comparing it with GSR and GyTAR. Our evaluation demonstrates that the proposed protocol surpasses GSR and GyTAR by over 20% in terms of packet delivery ratio, with the end-to-end delay reduced to less than 1.3 s on average.

## 1. Introduction

Intelligent transportation systems (ITSs) are garnering increasing attention globally due to the proliferation of vehicles and the emergence of various issues such as traffic congestion, air pollution, and traffic accidents. ITS integrates wireless and advanced IT technologies into mobile vehicles to enhance traffic safety and driver convenience. IEEE has introduced a new wireless communication standard called wireless access in vehicular environment (WAVE), based on IEEE 802.11p, to deliver these services. Consequently, research on vehicular ad-hoc networks (VANETs) is becoming increasingly active [1,2].

VANETs represent a next-generation network technology that facilitates vehicle-to-vehicle (V2V) and vehicle-to-infrastructure (V2I) communications and is a subclass concept of traditional mobile ad-hoc networks (MANETs). MANETs can establish independent ad-hoc networks among mobile nodes to construct autonomous networks. However, due to the mobile nodes’ limited characteristics, such as low bandwidth, low power, and limited resources, MANETs have limitations in realizing various services. In contrast, VANETs can support a broad spectrum of applications as vehicles are equipped with onboard units (OBUs) capable of robust processing without power limitations. These applications include safety measures like vehicle collision avoidance, emergency message dissemination, and traffic accident notification; driver convenience features like alternative route guidance, parking lot location, and gas station payment; and entertainment offerings such as games, movies, and music [3,4].

In VANETs, multi-hop routing protocols are crucial for supporting application services via wireless communication between vehicles. While various dynamic routing protocols for mobile networks have been proposed in existing MANET research [5,6,7,8], these protocols are unsuitable for VANETs. Designing a routing protocol for vehicular networks poses several challenges. Vehicles move rapidly, leading to frequent topology changes and network link breaks. Moreover, real-time variations in link quality occur based on vehicular traffic density. For instance, low traffic density results in higher packet delay due to fewer vehicles being available to relay packets, whereas high traffic density leads to lower packet delay. Urban environments can exacerbate these challenges by causing packet loss due to obstacles like tall skyscrapers interfering with radio signals. Therefore, vehicular routing protocols must address these demanding conditions diligently.

Greedy perimeter stateless routing (GPSR) stands out as a geographically based routing protocol deemed highly suitable for VANETs. It leverages GPS to identify nearby neighbors and employs greedy forwarding to direct packets to the node closest to the destination. GPSR is recognized for its speed, adaptability, and scalability, facilitated by its maintenance of a one-hop neighbor table. However, GPSR is primarily designed for highway scenarios, so it encounters frequent communication drops and high packet latency in urban scenarios [9]. Map-based geographic source routing (GSR) represents a junction-based geographic routing protocol that combines geographic-based (GPSR) and topology-based (DSR) protocols [10]. This method employs the vehicle’s digital map to establish a fixed sequence of junctions for routing packets to their destination. However, the junction sequence prioritizes the shortest distance to the destination, neglecting the traffic density through which the packets traverse. Conversely, the enhanced greedy traffic-aware routing protocol (GyTAR) integrates traffic awareness into the existing GSR protocol, introducing a junction-based geographic routing protocol [11]. GyTAR dynamically selects one junction at a time as traffic conditions evolve. The criterion for junction selection is based on the junction’s highest density of vehicle traffic and shortest distance to the destination among neighboring junctions. The GyTAR routing protocol has exhibited superior network performance compared to GSR.

With the rapid proliferation of vehicles in recent years, traffic monitoring systems have become an indispensable component of ITS. These systems can monitor sensor-equipped traffic vehicles for identification, speed, and traffic congestion in real-time, offering various applications. The smart traffic monitoring system (STMS) [12] is a traffic surveillance framework designed for monitoring traffic congestion and managing traffic lights. It operates as a fog node, gathering real-time data from geographically dispersed sensors and transmitting them to the cloud for storage and processing. Additionally, it can be adapted to detect traffic incidents that require immediate assistance amidst congested traffic. However, the STMS faces challenges due to the need to transmit the collected data to a base station, which can lead to bandwidth constraints, substantially impacting latency-sensitive applications. The infrastructure-free traffic information system (IFTIS) [13] offers a distributed mechanism for vehicles to collaborate in collecting traffic information on a road segment without relying on roadside units (RSUs). In this approach, the group leader of each cell in a road segment gathers traffic data and forwards them to the next junction using a greedy strategy. This technique offers substantial cost advantages as it eliminates the need for fixed infrastructure such as base stations. Moreover, it benefits from improved connectivity with an increase in the number of vehicles.

Recent research in vehicular networks has attempted to utilize artificial intelligence to address network latency and energy efficiency issues. In [14], they proposed an algorithm to improve QoS in vehicular networks by jointly scheduling deep neural network (DNN) inference tasks at the microarchitectural and network levels. This algorithm is a technique for making two-level scheduling decisions that utilizes deep reinforcement learning to respond to dynamic environments. The technique aims to minimize the total weighted sum of response time and energy consumption for all jobs under the constraints of response time, energy consumption, and storage capacity. In [15], an energy-efficiency secure offloading (EESO) technique based on asynchronous advantage actor–critic (A3C) was proposed in non-orthogonal multiple access (NOMA) offloading scenarios using physical layer security (PLS) techniques. This technique applies asynchronous deep reinforcement learning for highly dynamic automotive edge computing to reduce the energy consumed by the system and protect confidential information from eavesdropping. The centralized routing scheme with mobility prediction (CRS-MP), proposed in [16], introduces a centralized routing protocol for end-to-end unicast communication. By leveraging artificial neural networks (ANNs) to learn vehicle mobility patterns, the protocol offers a routing technique to improve packet delivery probability and minimize delay. Notably, the scheme does not necessitate continuous monitoring of vehicle mobility. Instead, it dynamically selects the routing path based on the probability of vehicle mobility, either by the software-defined networking (SDN) controller or the RSU/base station (BS).

Our study proposes multiple-junction-based traffic-aware routing (MJTAR), demonstrating lower latency and higher packet delivery success rates than existing traffic-aware geographic routing methods such as GyTAR [11]. MJTAR incorporates two fundamental mechanisms. First, it introduces an enhanced infrastructure-free traffic information system (E-IFTIS) mechanism to identify vehicle traffic from multiple junctions. E-IFTIS extends the capabilities of existing IFTIS [13] to collect vehicle traffic data for a road segment from up to two junctions. Second, MJTAR offers an optimal multiple junction selection scheme (OMSS) mechanism to determine the optimal packet path based on multiple junctions. OMSS utilizes an ant colony optimization algorithm [17] to compute the connectivity probability for each road segment between junctions. Subsequently, it dynamically selects multiple junctions (two-hop-based junctions) based on the connectivity probability of the road segments.

The research contributions of the MJTAR routing protocol are outlined as follows:

- We propose an optimal multiple junction selection scheme (OMSS) algorithm that utilizes the ACO algorithm to select the optimal multiple junctions. This algorithm employs a stochastic formula to explore the optimal multiple junctions by mimicking the behavior of biological ants.
- We present a distributed mechanism for estimating vehicle traffic density based on multiple junctions in a purely ad-hoc environment, eliminating the need for fixed infrastructure such as roadside units (RSUs).

The remainder of the paper is structured as follows: Section 2 briefly introduces the related work in this research area. Section 3 outlines the necessity of this work and introduces the MJTAR mechanism proposed in this study. Subsequently, Section 4 presents the performance evaluation based on extensive simulations using NS-3 [18] and SUMO [19] simulators. Finally, Section 5 concludes the thesis.

## 2. Related Work

### 2.1. Topology-Based vs. Geographic-Based Routing Protocols

Multi-hop wireless networks are commonly classified into topology-based and geographic-based routing [20]. Topology-based routing protocols can be reactive (on-demand), proactive (table-driven), or hybrid. Reactive routing protocols (such as DSR and AODV) maintain routing paths that are currently in use. On the other hand, table-driven routing protocols (like OLSR—optimized link state routing protocol) uphold all the routing paths in the network topology. Hybrid routing protocols (e.g., ZRP) combine the strengths of both on-demand and table-driven routing protocols. Protocols such as on-demand and table-driven require maintenance of the entire network topology and routing table information of all nodes for packet forwarding [21]. When applied to vehicular networks, specific protocols are deemed unsuitable for VANETs due to the dynamic nature of vehicle communication and vehicles’ high speed and mobility, which can result in elevated communication congestion. However, geographic routing protocols [21,22] are regarded as highly adaptable to high speeds and mobility, making them suitable for VANETs. Greedy perimeter stateless routing (GPSR) [9] is a prominent geographically based routing protocol in VANETs. GPSR employs two routing strategies for packet forwarding: greedy forwarding and perimeter forwarding. The greedy forwarding strategy directs packets to neighboring nodes closest to the destination, gradually moving them toward the target. If, during forwarding, no neighbor is closer to the destination than the current node, it encounters a local maximum. In such cases, the perimeter forwarding strategy is utilized to navigate on a right-handed basis. GPSR demonstrates high adaptability and scalability by maintaining a one-hop-based table. However, while it exhibits excellent routing performance on highways, it often encounters greedy forwarding failures in environments due to various obstacles like buildings, trees, and other impediments. Consequently, perimeter forwarding is frequently employed, increasing hop count and elevating end-to-end delay.

### 2.2. Junction-Based Routing Protocols

**Figure 1:**
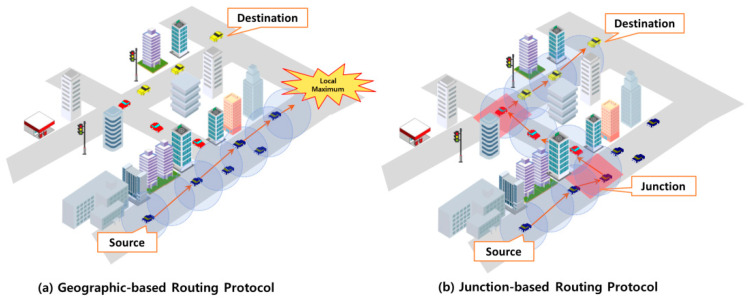
The need for junction-based routing protocol in urban environments.

The design of VANET routing protocols in urban environments necessitates the consideration of many complex factors. Urban areas, such as those with tall buildings and trees, impede vehicle wireless communication. The junction-based routing protocol [23] addresses the limitations of geographically based routing protocols in urban settings. It proposes a routing protocol that leverages junction points devoid of radio interference to forward packets to their destinations efficiently, as shown in Figure 1.

GSR [10] is a VANET routing protocol tailored explicitly for urban environments. A junction-based geographic routing protocol integrates geographic-based (GPSR) and topology-based (DSR) techniques. GSR utilizes the digital map in the vehicle’s navigation system to locate junctions and determines the sequence based on the shortest distance to the destination using Dijkstra’s algorithm. Packets generated by the source are then forwarded through the junction sequence to the destination. However, this approach overlooks traffic density considerations, potentially resulting in routing paths with low connectivity. Greedy perimeter coordinator routing (GPCR) [23] adopts a limited greedy mode for forwarding packets. When selecting the next forwarding node around a junction, it prioritizes coordinator nodes (nodes at the junction) over non-coordinator nodes closer to the destination. If the event encounters a local maximum, it enters recovery mode and forwards packets counterclockwise, following the right-hand rule. However, this recovery mode increases end-to-end delay due to unnecessary relay nodes and imposes a high overhead due to the large number of beacon messages. Also, this routing protocol does not consider traffic awareness.

### 2.3. Traffic-Aware Routing Protocols

Since network performance in VANETs is highly dependent on vehicle traffic density, routing protocols that include junction selection techniques that take traffic density into account are essential. Previous works [24,25,26,27] proposed an optimal junction selection technique that considers traffic conditions at junctions. This technique uses fog nodes in the RSU to collect traffic information to select the optimal junction considering traffic flow, traffic density, and vehicle speed. However, this technique can have a devastating impact on delay-sensitive applications as the traffic bandwidth of the RSUs rapidly decreases as the number of vehicles around the intersection increases. Furthermore, the need to deploy RSUs at each junction is cost prohibitive. In [11], a traffic-aware optimal junction selection technique based on traffic awareness was proposed by collecting vehicle information without the help of RSUs in a vehicular ad-hoc environment. This approach circumvents the selection of junctions (anchors) because even if the distance to the destination is close, the network will perform poorly on roads with low vehicle traffic density. Conversely, it acknowledges that roads with high vehicular traffic density exhibit superior network performance, thus opting for those junctions to forward packets to the destination [20,28].

Anchor-based street- and traffic-aware routing (A-Star) [29] is a traffic-aware routing protocol that leverages city bus routing data. The rationale is to utilize city bus routes to determine the sequence of junctions (anchors) since these routes typically experience high vehicle traffic density. However, as this technique solely focuses on traffic density, the resulting routing path may not be optimal.

GyTAR [11] dynamically selects a junction by considering the optimal distance and traffic density for packets to reach their destination. GyTAR employs the infrastructure free-traffic information system (IFTIS) to estimate traffic density between junctions without relying on fixed infrastructure such as RSUs. The forwarding vehicle utilizes the navigation system’s road map to locate neighboring junctions. For each candidate neighborhood junction, the forwarding vehicle computes the shortest distance to the destination [30] and assigns a score based on vehicle traffic density weight. Subsequently, the junction with the highest score among the candidate neighboring junctions is selected as the next neighbor junction, and the packet is forwarded. Suppose a forwarding node encounters a local maximum between junctions. In that case, it employs a recovery mode by executing “carry and forward” [31], where packets are stored in a buffer and forwarded upon encountering a nearby vehicle or reaching the next junction. However, as this protocol only considers traffic density for the first neighboring junction, the traffic density between the next two neighboring junctions remains unpredictable. In other words, the subsequent selection of the second neighboring junction may occasionally encompass roads without vehicular traffic. Ultimately, this scenario can result in a diminished packet delivery ratio due to packet loss or increased end-to-end delay stemming from frequent recovery strategies. The following Table 1 describes the characteristics and comparison of MJTAR proposed in this work and previously proposed vehicular routing protocols.

### 2.4. ACO-Based Routing Protocol

In combinatorial optimization, ant colony optimization (ACO) [17] emerges as a prominent swarm intelligence approach inspired by the foraging behavior of ants. As ants explore paths near their nest, they release chemicals known as pheromones. These pheromones serve as cues for other ants to navigate back to the next. Moreover, when an ant discovers a new food source, it leaves a trail of pheromones while transporting the food back to the nest, enabling other foraging ants to follow the scent and locate the food source. However, these pheromones are volatile and evaporate over time. Ants tend to favor paths with stronger pheromone odor among multiple paths, allowing them to identify the shortest route from the nest to the food source. ACO operates as a heuristic algorithm to obtain effective solutions to challenging combinatorial optimization problems within a reasonable computational time.

In prior research on VANET routing protocols, ACO has been utilized to discover optimal routes. VACO (vehicular routing protocol based on ant colony optimization) [32,33] was introduced to assess the relay quality of road segments between junctions within an urban environment. This protocol operates under the assumption that RSUs are deployed at each junction to facilitate the identification of the optimal packet path. The source node dispatches multiple forward ants toward the target RSU nearest the destination to establish a route. Subsequently, backward ants gather relay quality information for each road segment and relay it back to the source node through the designated target RSU. The source node can then aggregate data from the RSUs to determine the optimal route regarding latency, bandwidth, and delivery ratio. However, this protocol entails substantial installation costs, necessitating an RSU at every junction. Moreover, the innate characteristics of ants are forfeited in the event of RSU failure.

MAR-DYMO (mobility-aware ant colony optimization routing DYMO) [34] represents an enhancement over the existing DYMO (dynamic MANET on-demand routing) [35] by introducing an ant-based routing algorithm capable of predicting route lifetime through the utilization of position and speed information of vehicles. Each vehicle determines a phenomenon level that forecasts route lifetime by considering the position, speed information, and the likelihood of message reception. Based on these pheromone levels, The source node determines the optimal route to the destination node. After the path lifetime, an evaporation mechanism is implemented to dissipate the pheromone completely. However, this mechanism suffers from limitations such as high overhead and limited scalability, mainly when the source node must discover a new route following a link failure.

## 3. MJTAR Protocol

### 3.1. Hypothesis

**Figure 2:**
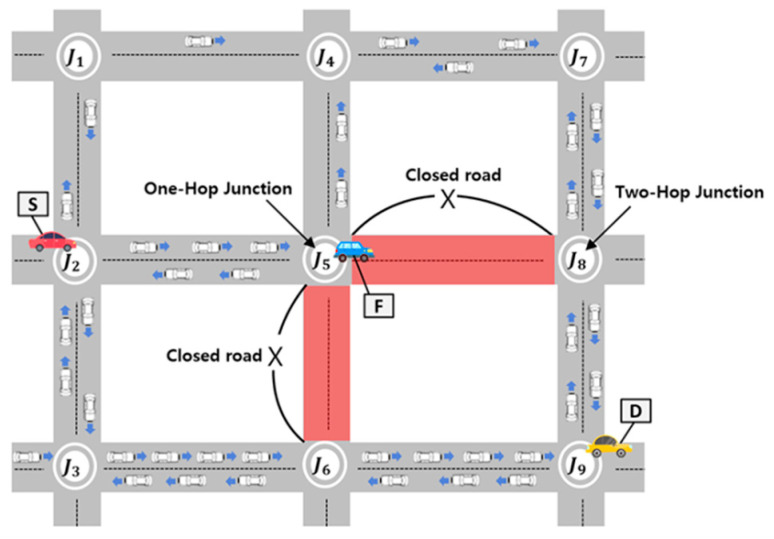
Traffic-aware VANET scenario.

To discuss the limitations of the existing GyTAR protocol, let us consider the scenario depicted in Figure 2 below. In this illustration, the solid lines denote a two-lane road in both directions, with vehicles moving in the direction of each arrow. Here, S represents the source, D denotes the destination, and F signifies a forwarding vehicle responsible for relaying packets. Let us assume that the source vehicle located at junction $J_{2}$ is tasked with forwarding packets to the destination.

Dynamic junction-selection-based routing protocols, such as GyTAR [11], are designed to determine the next neighboring junction based on traffic density and the shortest distance to the destination. Consequently, a source vehicle employing this protocol would opt for $J_{5}$ to forward a packet. However, upon receiving the packet, the forwarding vehicle stationed at $J_{5}$ encounters an unforeseen situation. The forwarding vehicle at $J_{5}$ intends to select $J_{8}$ to relay the packet toward its destination. Unfortunately, the road between $J_{5}$ and $J_{8}$ is a closed, devoid of vehicular traffic. Consequently, encountering this closed road leads to a local maximum, resulting in heightened end-to-end delay or even packet loss. Conversely, the road between $J_{5}$ and $J_{6}$ similarly presents a closed road scenario. Ultimately, this limitation in the existing protocol may result in the selection of an inefficient routing path despite the optimal path ($J_{2}$-$J_{3}$-$J_{6}$-$J_{9}$). To address these challenges, we propose the MJTAR protocol. This dynamic multiple-junction-based routing protocol considers traffic density and the shortest distance to the destination up to two junctions at a time. This protocol aims to mitigate packet delay and enhance the packet delivery success ratio by assessing the vehicular traffic density between two junctions. However, our proposed MJTAR also exhibits certain limitations. If vehicles at junctions consider traffic from up to two junctions, it entails a higher computational effort than previous approaches. However, this compromise is necessary to forecast closed roads effectively. Extending the consideration of traffic to 3 or 4 junctions instead of 2 would result in an increased overhead for route discovery and a decline in network performance. Therefore, in this study, we constrain the consideration of junctions to a maximum of two.

### 3.2. MJTAR Overview

Our proposed MJTAR protocol incorporates two fundamental mechanisms: (1) the E-IFTIS mechanism, capable of estimating traffic density up to two-hop junctions; (2) the OMSS applies the ACO algorithm to select the optimal multiple junctions.

MJTAR uses a GPS receiver to determine its position and speed. Additionally, it leverages the digital map provided by the vehicle’s navigation system to ascertain the position of neighboring junctions and acquire road-level information. For the source vehicle to determine the position of the destination vehicle, GLS [36] is employed, utilizing a network of wireless sensors in urban environments to discover the destination’s position. Furthermore, each vehicle is assumed to maintain a neighborhood table containing its neighbors’ position, speed, and direction. This table is constructed based on all vehicles’ periodic Hello (beacon) packet transmission. Our proposed MJTAR draws inspiration from the modified version of ant colony optimization (MACO) [37] and applies the ACO algorithm. MACO is an algorithm that mimics the behavior of artificial ants on their way to work, recognizing congestion when encountering a strong concentration of pheromones and consequently seeking less-congested routes. In contrast, MJTAR’s algorithm identifies high pheromone concentration as indicative of high traffic density and navigates routes with strong wireless connectivity. Subsequently, we define a probabilistic algorithm capable of selecting the optimal multiple junctions using the weight of the shortest distance to the destination in conjunction with the permeant and the destination.

### 3.3. E-IFTIS Overview

The enhanced infrastructure-free traffic information system (E-IFTIS) is a fully distributed mechanism designed to enhance the existing approach for estimating vehicle traffic density. It extends the capability of the current mechanism, IFTIS [13], which estimates vehicle traffic density up to the first neighbor junction (one-hop junction). 

#### 3.3.1. IFTIS

**Figure 3:**
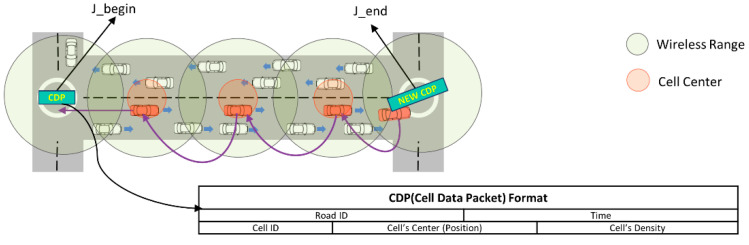
Infrastructure-free traffic information system mechanism.

The infrastructure-free traffic information system (IFTIS) is a distributed mechanism designed to estimate vehicle traffic without relying on fixed infrastructure. Within IFTIS, each roadway, defined as a segment of street between two junctions, is subdivided into cells of a certain size. Vehicles within each cell are then grouped into a single entity. This organizational structure facilitates the efficient assessment of traffic conditions and allows for estimating vehicle density and movement patterns along the roadway segment. The following Figure 3 illustrates this grouping process:

By employing this approach, IFTIS enables real-time vehicle traffic estimation, providing valuable information for traffic management and routing optimization without any fixed infrastructure.

The cell size can vary depending on the transmission range of the vehicle, with the vehicle closest to the cell center (the one traveling in the direction of the J_end junction) designated as the cell group leader. As the group leader exits the road and reaches the J_end junction, it generates a new cell data packet (CDP) and forwards it in a greedy forwarding fashion toward the J_begin junction. Meanwhile, intermediate group leaders gather vehicle information from neighboring vehicles within the same cell, facilitated by periodic transmission of Hello packets. Upon nearing the J_begin junction, the CDP is disseminated to neighboring vehicles, allowing those at the J_begin junction to estimate the traffic situation up to the one-hop junction (J_end) without relying on fixed infrastructure. In our research, we introduce an E-IFTIS mechanism, which extends the existing mechanism to estimate vehicle traffic to two-hop junctions. Building upon E-IFTIS, we propose a more effective dynamic multiple junction selection technique.

#### 3.3.2. E-IFTIS Concept

**Figure 4:**
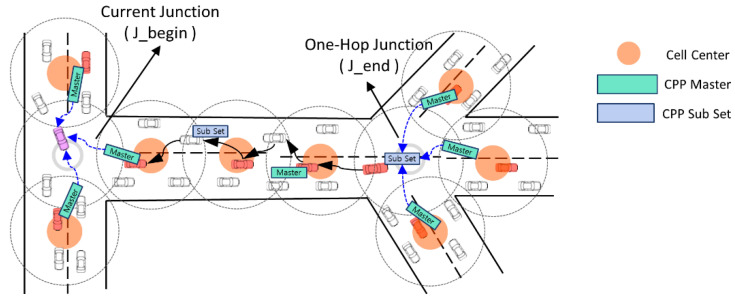
Concept of the enhanced IFTIS distributed mechanism.

The following Figure 4 illustrates the operational concept of the proposed E-IFTIS.

**Figure 5:**
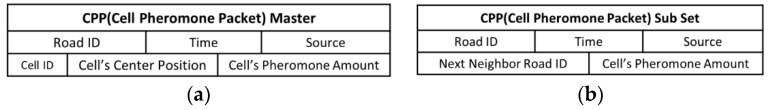
CPP Packet Format. (**a**) CPP Master Packet Format; (**b**) CPP Subset Packet Format.

In our enhancement, we introduce an additional field to the existing CDP to accommodate pheromone information, transforming it into a cell pheromone packet (CPP). We posit that the quantity of pheromone contained within the CPP correlates with the number of vehicles on the road. Consequently, group leaders traversing the road collect pheromone data from neighboring vehicles within the same cell and incorporate them into the CPP for storage and transmission. The following Figure 5 describes the CPP packet format.

The CPP is divided into two structures: CPP master and CPP subset. The CPP master structure serves as a packet to store the pheromone collected from each cell and transmit it to the J_begin junction. It encompasses fields for identifying the road ID, transmission time, cell’s center position, and a list detailing the pheromone amount within the cell. On the other hand, the CPP subset structure functions as an agent packet responsible for consolidating CPP master packets received from multiple roads at the one-hop junction into a unified group. Subsequently, these consolidated data are forwarded to the J_begin junction. Upon reaching the J_begin junction, the vehicle collects both the CPP master and CPP subset packets. Subsequently, it utilizes the gathered information to populate the CPP management table, storing details regarding junction locations and pheromone information. Nevertheless, it cannot gather CPPs during early morning hours when vehicle density is low or in road conditions devoid of moving vehicles. In such scenarios, a hybrid approach may be suggested, wherein E-IFTIS is employed during peak traffic periods in the morning or evening when vehicle density is high, while RSU traffic surveillance systems [12,38,39,40] are utilized during off-peak hours. The subsequent sections will elaborate on the CPP creation procedure and the method for maintaining CPP in detail.

#### 3.3.3. New CPP Generation Procedure

**Figure 6:**
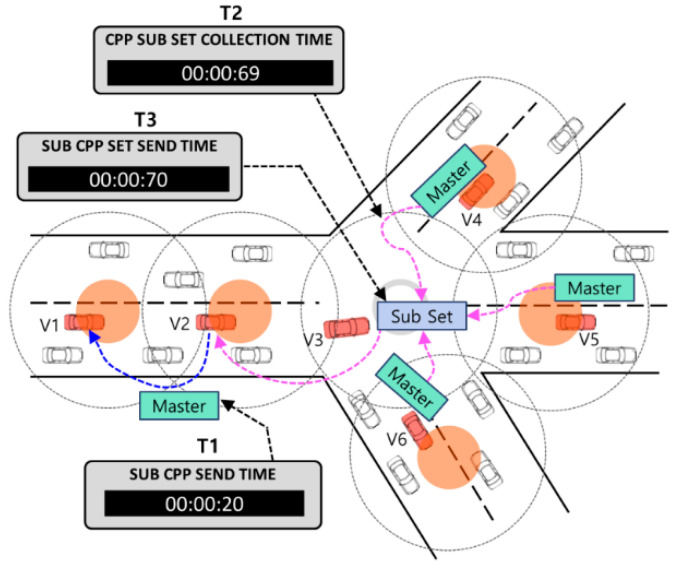
CPP generation procedure at one-hop junction.

Figure 6 illustrates the initial creation process for CPP master and CPP subset packets.

When group leader V3 exits the road and reaches a one-hop junction, it generates a new CPP master packet. It transmits it to group leader V2 via grid forwarding. Group leader V2 gathers pheromone data from neighboring vehicles within the same cell and stores them within the CPP master. Subsequently, group leader V2 forwards the CPP master to the subsequent group leader, V1. Meanwhile, group leader V3, the originator of the CPP master, promptly generates a CPP subset packet. Upon its creation, group leader V3 collects CPP master packets from the connected roads within a 0.5 s timeframe and integrates them into the CPP subset. Subsequently, group leader V3 employs greedy forwarding to transmit the CPP subset packet to group leader V2. Ultimately, the CPP subset is an agent packet tasked with aggregating multiple CPP masters and delivering them to the J_begin junction.

#### 3.3.4. CPP Collection Procedure

**Figure 7:**
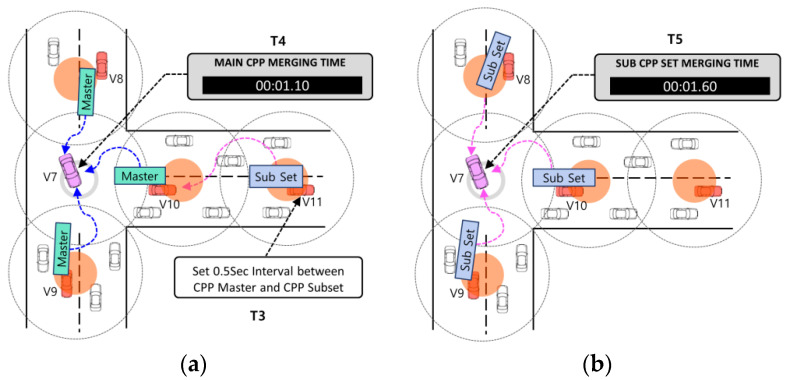
(**a**) Collect CPP master information during 0.5 s. (**b**) Collect CPP subset information during 0.5 s.

Figure 7 shows how to collect CPP master and subset packets and manage CPP information for vehicles near the J_begin junction.

Upon vehicle V7’s entry into the current junction, a timer initiates, prompting it to gather CPP master and CPP subset packets within a 0.5 s interval. Initially, V7 retrieves the CPP master from the connected road, as depicted in panel (a) of Figure 7. Subsequently, it gathers multiple CPP subsets from subsequent roads, illustrated in panel (b) of Figure 7. If no CPP master or CPP subset packets are received from a specific road within the designated timeframe, it is deemed a low-traffic road and disregarded. V7 ceases the collection of CPP master and CPP subset packets upon the timer’s expiration. Based on the collected CPP data, the junction location and pheromone information are updated within the CPP management table. Each vehicle maintains its own CPP management table for reference. 

**Figure 8:**
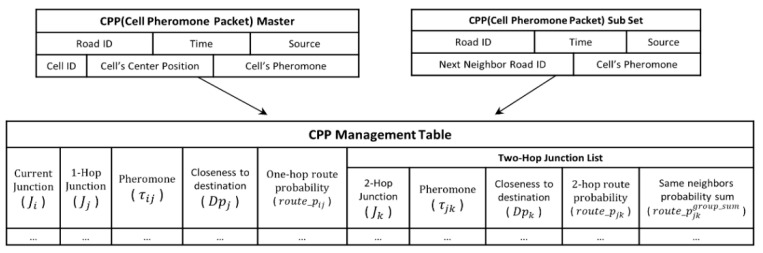
The definition of the CPP management table.

The rationale behind the independent implementation of E-IFTIS between vehicles, without reliance on RSUs as in [26], stems from the assumption that vehicles are equipped with digital maps and GPS receivers. Algorithm 1 outlines the procedure for collecting CPPs at junctions. On the road, the group leader forwards the collected CPP packets to the subsequent junction utilizing a greedy forwarding strategy. Upon receipt of a CPP packet by the last group leader or a neighboring node close to the junction, it broadcasts the packet to its passing neighbors. Lines 3 through 11 ascertain whether the vehicle is approaching the junction location via its GPS receiver. Upon arrival at the junction, the vehicle acquires the CPP master packet, initiating the timer. Subsequently, within the time limit, the vehicle receives the CPP master and CPP subset packets, updating the collected CPP information to the CPP management table, as illustrated in Figure 8. The delivery rate of CPP packets may fluctuate based on the road traffic density. In line 14, the FindOptimizedPath function is invoked, utilizing the CPP management table to determine the optimal path using Equations (1)–(9).
**Algorithm 1:** Collect CPP at junctions**Input:** $CurrPos_{GPS}$, $Junc_{Pos}$, $Packet_{CPP}$, $CPP\_Management_{table}$ **Output:** void**Begin**$Timer_{collection}$ ← null**if** $CurrPos_{GPS}$ = $Junc_{Pos}$ **then**  **if** $Packet_{CPP}.\mathrm{Type}$ = CPP_Master **OR** CPP_Sub_Set **AND then** 
     $Timer_{collection}$ ← GetTimerStart(1 s)       **while** $Timer_{collection}$ ≠ TIMEOUT **do**          **if** $Packet_{CPP}.\mathrm{Type}$ = CPP_Master **then**                $CPP\_Management_{table}$.Update($Packet_{cpp}$)          **else if**  $Packet_{cpp}.\mathrm{Type}$ = CPP_SubSet **then**                $CPP\_Management_{table}$.Update($Packet_{cpp}$)          **else if**          **if** $CPP\_Management_{table}$ ≠ null **then**               **if** $CurrPos_{GPS}$ = $Junc_{Pos}$ **then**                  FindOptimizedPath($CPP\_Management_{table}$)               **end if**            **end if**          **end while**   **end if****end if**return 0

Table 2 presents the notation to differentiate the fields configured within the CPP management table.

The CPP management table maintains a list for identifying the current junction and 1-hop candidate junctions. Each entry in the list includes the following fields for every 1-hop candidate junction: Junction ID, pheromone amount, shortest distance to the destination, and a two-hop junction list. The 2-hop junction list comprises a record of junction IDs and corresponding pheromone information for the 2-hop candidate junctions neighboring the 1-hop candidate junctions.

#### 3.3.5. CPP Management Table Update Procedure

This section presents a method for vehicle V7 to update the value of each field configured in the CPP management table after the timer ends. Initially, the amount of pheromone ($\tau_{ij}$) for a 1-hop candidate junction is determined according to Equations (1)–(4). In Equation (1), $N_{avg}$ represents the average pheromone value in each cell. $N_{c}$ denotes the number of cells between $J_{i}$ and $J_{j}$, while $N_{i}$ signifies the amount of pheromone in one cell. For this study, it is assumed that the amount in each vehicle is uniform and set to 1.

(1) $$\left(N_{avg}=\frac{1}{N_{c}}*\;\sum_{i=1}^{N_{c}}N_{i}\right)\;\;$$

In Equation (2), σ is the standard deviation of the amount of pheromone distributed over all cells between $J_{i}$ and $J_{j}$. The standard deviation indicates how far away it is from $N_{avg}$. A large standard deviation indicates that the cell density is far from the mean, while a small standard deviation indicates that the cell density is clustered closely around the mean.

(2) $$\sigma =\sqrt{\left(\frac{1}{N_{c}}*\left(\sum_{i=1}^{N_{c}}{\left(N_{i}-N_{avg}\right)}^{2}\;\right)\right)}$$

In Equation (3), $\tau_{\mathrm{ij}}^{v}$ represents the formula for calculating the total pheromone amount between $J_{i}$ and $J_{j}$. $N_{con}$ denotes the theoretical pheromone constant for the cell, set to 12 in this study. ϕ represents an expression mimicking the evaporation effect of pheromone over time, as illustrated in Equation (4). Consequently, when a CPP packet reaches the current junction (*J_i_*), it undergoes a penalty proportional to its experienced delay, reducing the pheromone amount.

(3) $$\tau_{\mathrm{ij}}^{v}=\min\left[\left\{\left(1-\phi \right)*\;\frac{1}{\sigma +1}*\;\frac{N_{\mathrm{avg}}}{N_{\mathrm{con}}}\right\},1\right]$$

(4) $$\phi =\;packet\_recv\_time_{cpp\_master}-packet\_send\_time_{cpp\_master}$$

The pheromone amount ($\tau_{jk}$) for the 2-hop candidate junction is determined using Equations (1)–(4) in a similar manner. The proximity to the next destination ($Dp_{j})$ can be computed utilizing Equations (5) and (6). The following notation is defined to facilitate the calculation of the proximity to the next destination.

$D_{i}$, $D_{j}$, and $D_{k}$ can obtain the curve metric [22] distance to the destination using the following Equation (5).

(5) $$d_{j}\left(x,y\right)=\sum_{j=1}^{m}\left|\;x_{j}-y_{j}\;\right|$$

(6) $$Dp_{j}=D_{j}/D_{i}$$

(7) $$Dp_{k}=D_{k}/D_{i}$$

The proximity ($Dp_{j}$) between the 1-hop candidate junction and the destination is determined using Equation (6), while the proximity ($Dp_{k})$ between the 2-hop candidate junction and the destination is calculated using Equation (7).

### 3.4. OMSS Overview

**Figure 9:**
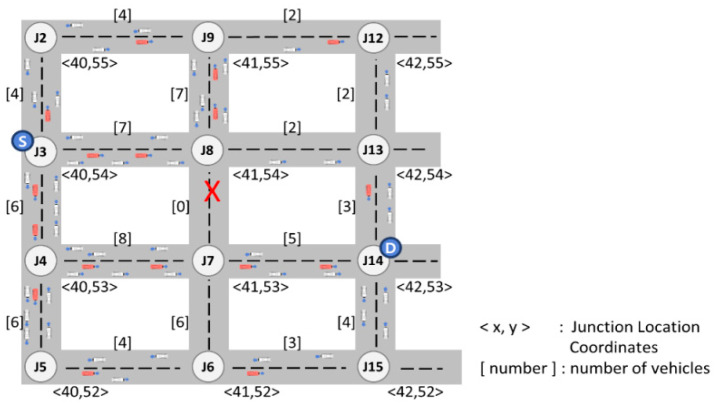
An example scenario of urban streets.

The optimal multi-junction selection scheme (OMSS) introduced in this study is an algorithm designed to explore 2-hop junctions with highly connected roads using ACOs probabilistically. Figure 9 presents an example scenario to validate the OMSS algorithm’s feasibility.

In Figure 9, <x,y> denotes the junction location, and [number] indicates the number of vehicles traveling on each road. Let us consider that the source vehicle at J3 initiates the packet and forwards it to the destination vehicle at J14. The subsequent Table 3 illustrates an example of updating the junction ID, the pheromone amount, and the proximity to the destination for a 1-hop candidate junction and a 2-hop candidate junction. These computations are performed using Equations (1)–(8), followed by their update to the CPP management table.

#### Junction Route Probability Procedure

The source vehicle can compute the probability values for the 1-hop candidate junction and the 2-hop candidate junction using Equation (8). This calculation involves referencing the pheromone amount stored in the CPP management table and the proximity to the destination.

(8) route_pijt=τijt]α * 1Dpj]β∑l∈allowedv τijt]α* 1Dpj]β      if ij ∈ allowedv 0                                                     Otherwise

$\tau_{ij}\left(t\right)$ represents the amount of pheromone collected at time *t*, and $\frac{1}{Dp_{j}}$ is the inverse of the proximity to the destination. α serves as a weighting factor for the pheromone amount, while β functions as a weighting factor for the proximity to the destination. In this study, the weights of both α and β are set to 1. Table 4 illustrates an example of the source vehicle updating the probability values for each 1-hop candidate junction and 2-hop candidate junction using Equation (8).

The probability value increases with a relatively higher amount of pheromone and a lower distance weight to the destination. The 1-hop candidate junction, J8, has the highest probability of 0.4667. Meanwhile, the 2-hop candidate junction, J9—a neighbor to the 1-hop candidate junction—has the highest probability of 0.1489. However, the location of J9 is opposite from the destination, leading to increased packet latency. To address this issue, we group the probability values of 2-hop candidate junctions that share the same neighborhood relationship as 1-hop candidate junctions. Subsequently, we sum them up and store the result in $route\_p_{jk}^{group\_sum}$. Then, we calculate the probability average of $route\_P_{ij}$ and $route\_p_{jk}^{group\_sum}$, as shown in Equation (9).

(9) $$p\_avg_{best}=\frac{selected\_p_{ij}\left(t\right)+route\_p_{jk}^{group\_sum}\;}{2}$$

The values of the fields $J_{i}$, $J_{j}$, $J_{k}$, $route\_P_{ij}$, and $route\_p_{jk}^{group\_sum}$ are stored separately in the ant metrics table, along with the calculated average ($p\_avg_{best}$), as show in the following Table 5. Ultimately, the source vehicle selects the 1-hop junction and the 2-hop junction with the highest probability ($p\_avg_{best}$) from the ant metrics table.

**Figure 10:**
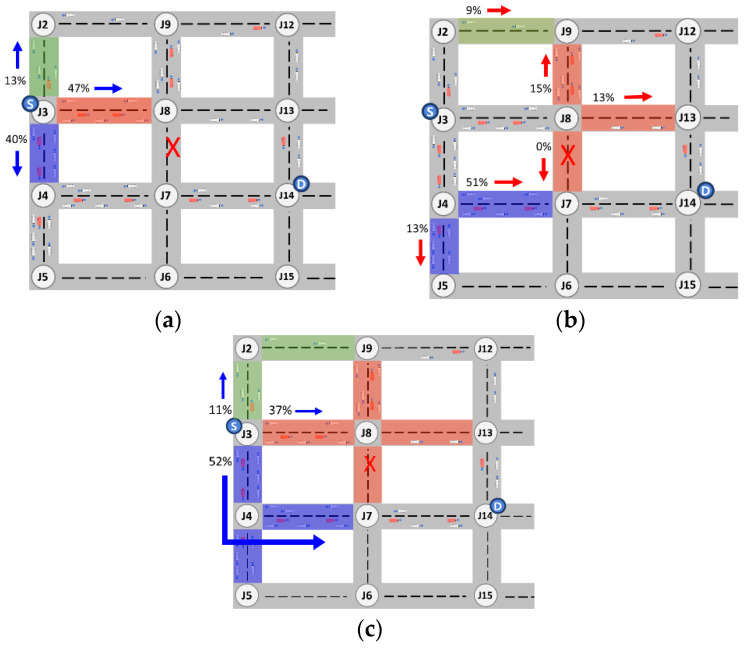
Concept of dynamic multiple junction selection technique applying ant colony algorithm. (**a**) 1-hop junction selection probability; (**b**) 2-hop junction selection probability; (**c**) Average probability of choosing a 2-hop junction; (X: No vehicle traffic).

Hence, the source vehicle will employ the OMSS algorithm to opt for the J4–J7 junction, which exhibits the lowest latency and the highest probability of successful packet delivery, as depicted in Figure 10.

## 4. Performance Evaluation

In this section, we conduct simulations using the NS-3 simulator to assess the performance of MJTAR. Additionally, we implement the GSR [10] and GyTAR [11] protocols previously designed for urban settings in NS-3. As the first vehicular ad-hoc routing protocol tailored for urban environments, GSR forwards packets by computing the junction sequence based on the shortest distance from the source to the destination. The choice of mobility model substantially influences protocol behavior and simulation results. Hence, we employ the SUMO simulator for the mobility model and integrate NS-3 with SUMO for the simulations. This section describes the simulation environment in Section 4.1 and details the performance analysis of packet delivery ratio, end-to-end delay, and byte overhead in Section 4.2.

### 4.1. Simulation Setup

**Figure 11:**
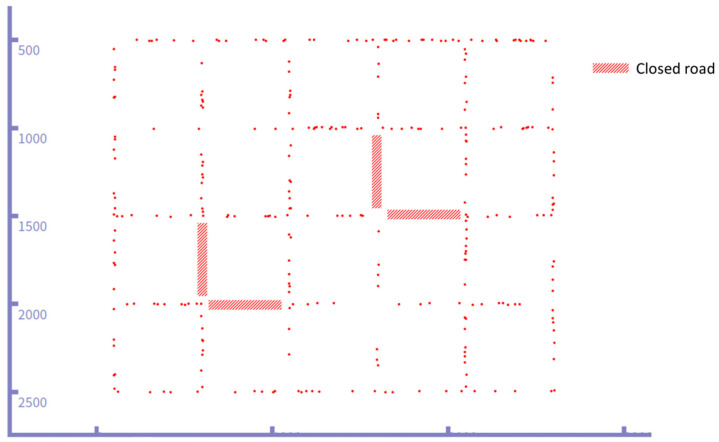
Simulation of VANET topology.

The simulation scenario encompasses a rectangular street area measuring 3000 × 2400 $m^{2}$ and features 30 junctions interconnected by 49 multi-lane, two-way roads, as illustrated in Figure 11 below. Each road is populated with randomized vehicles, with only one designated as the source vehicle. The source vehicle traverses the road randomly, dispatching packets to the destination as it moves. The destination is at one of the junctions, while the two rectangles marked with a diagonal pattern represent closed roads inaccessible to vehicles.

The simulation parameters used for performance evaluation are outlined in Table 6.

### 4.2. Simulation Results and Analysis

The proposed routing and other traffic-aware routing protocols, GSR and GyTAR, are evaluated based on the following metrics:

- Packet delivery ratio: the percentage of packets successfully delivered from the source to the destination.
- End-to-end delay: the time it takes for packets to traverse the network from the source to the destination.
- Bytes overhead is the percentage of total control packet bytes incurred before the simulation fully runs, and the packet arrives at its destination; it is calculated by accumulating the number of bytes in control packets.

#### 4.2.1. Packet Delivery Ratio

**Figure 12:**
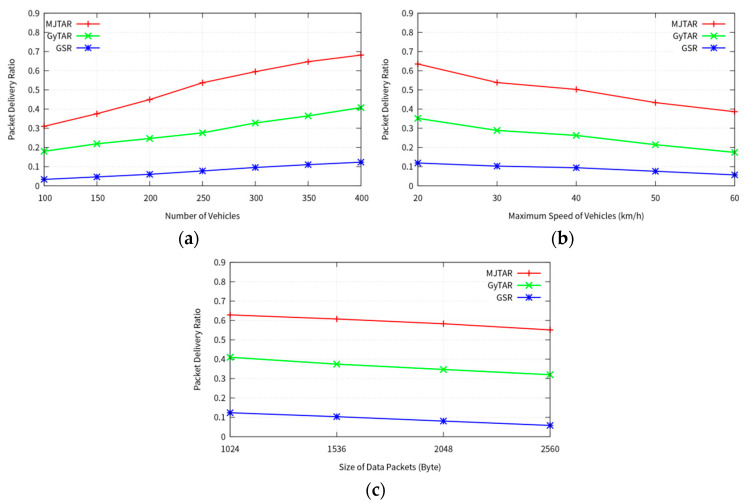
Comparison between proposed GSR, GyTAR, and MJTAR for packet delivery ratio; (**a**) number of vehicles (5 packets/s); (**b**) speed of vehicles (300 nodes and 5 packets/s); (**c**) packet size (300 nodes and 5 packets/s).

Figure 12a illustrates the comparison of packet delivery ratios as a function of the number of nodes. It is evident that all protocols exhibit a higher packet delivery ratio as the number of nodes increases, with MJTAR demonstrating the highest packet delivery ratio among the protocols. This superiority can be attributed to MJTAR’s multiple-junction-based traffic awareness mechanism, enabling it to avoid closed roads and select routes with higher connectivity. Conversely, GyTAR displays a lower packet delivery ratio than MJTAR, primarily because it assesses traffic one junction at a time, increasing the probability of encountering closed roads. Figure 12b presents the comparison of packet delivery ratios based on node movement speed. As the node travel speed increases, the packet delivery ratio decreases. This phenomenon occurs due to the rapid changes in vehicle positions. The greedy forwarding technique selects the node closest to the destination for packet forwarding. However, as node speed increases, these selected nodes may move out of range more quickly, leading to increased packet loss.

Figure 12c depicts the packet delivery ratio for various packet sizes at a node traveling speed of 30 km/h. MJTAR maintains the highest packet delivery ratio compared to other protocols across different packet sizes. However, as the packet size increases, the ratio decreases. This decline can be attributed to the increased likelihood of packet collisions and fading as the packet size grows.

#### 4.2.2. End-to-End Delay

**Figure 13:**
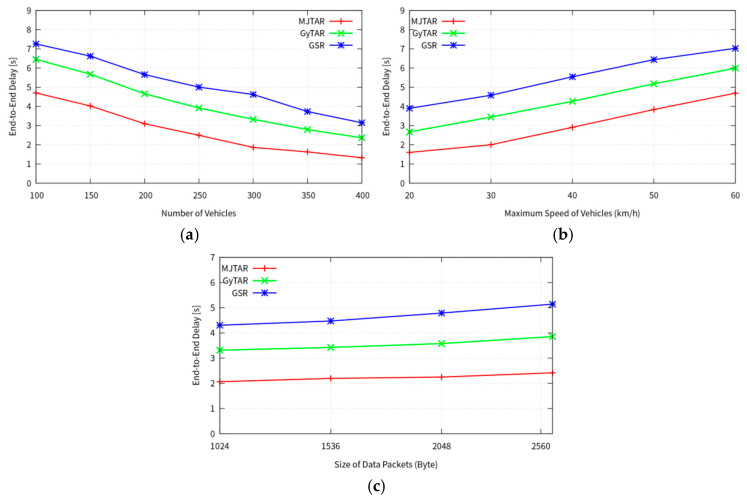
Comparison between proposed GSR, GyTAR, and MJTAR for end-to-end delay; (**a**) number of vehicles (5 packets/s); (**b**) speed of vehicles (300 nodes and 5 packets/s); (**c**) packet size (300 nodes and 5 packets/s).

Figure 13a illustrates the end-to-end delay concerning the number of nodes. MJTAR exhibits the lowest end-to-end delay compared to other protocols. This result is attributed to MJTAR’s ability to leverage higher connectivity stemming from increased traffic density as the number of nodes rises. Figure 13b plots the end-to-end delay against vehicle travel speed. Two factors contribute to higher delays with increased vehicle speeds. First, higher vehicle speeds result in shorter durations spent at junctions. This phenomenon occurs because elevated speeds heighten the likelihood of missing a Hello packet between neighboring vehicles at a junction, the data packet is buffered, and the junction selection mechanism is executed at the subsequent junction. Second, vehicle speeds exceeding the neighbor table update rate exacerbate delays. Specifically, when a vehicle forwards packets to a neighbor that may overtake it in the opposite direction, temporary looping can occur as the overtaking process unfolds. This temporary looping phenomenon contributes to increased delays.

Figure 13c depicts the end-to-end delay for various packet sizes while nodes travel at 30 km/h. Interestingly, MJTAR demonstrates that as the packet size increases, the end-to-end delay does not substantially change despite the occurrence of packet collisions.

#### 4.2.3. Bytes Overhead

**Figure 14:**
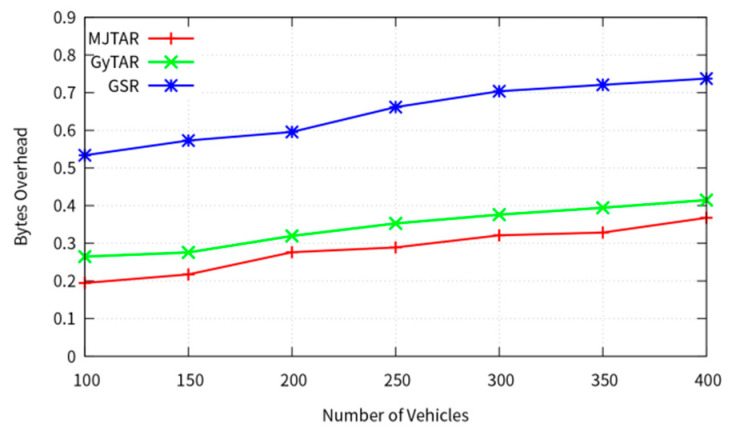
Bytes overhead.

In Figure 14, we assess the routing overhead of the three protocols relative to vehicle density. The routing overhead is quantified in bytes and is computed as the cumulative sum of control packet bytes generated between relay nodes on a road segment. It is expressed as a ratio by comparing the data packet delivery ratio to the size of the control packet bytes. A lower byte count signifies reduced overhead.

(10) $$\mathrm{Bytes}\;\mathrm{Overhead}=\frac{\mathrm{Hello}\;\mathrm{Packet}+\mathrm{Unicast}\;\mathrm{Routing}\;\mathrm{Control}\;\mathrm{Pakcet}+\mathrm{Data}\;\mathrm{stored}\;\mathrm{in}\;\mathrm{buffers}}{\mathrm{Data}\;\mathrm{packet}\;\mathrm{delivery}\;\mathrm{rate}}$$

As depicted in Figure 14, all three protocols exhibit a proportional increase in the percentage of control packets generated with the number of nodes. However, MJTAR demonstrates lower routing overhead than the other protocols. GSR tends to incur higher routing overhead due to its increased generation of Hello packets to gather neighbor position information. Conversely, MJTAR minimizes routing overhead by exclusively acquiring position information within its cell area through the E-IFTIS mechanism. Moreover, GSR’s tendency to select roads with lower traffic density, in contrast to the MJTAR protocol, leads to a higher frequency of Hello packets due to frequent recovery mode activations. Consequently, the frequency of Hello packets generated by GSR is approximately three times higher than that of MJTAR.

## 5. Conclusions

This study introduces the MJTAR protocol tailored for urban vehicular ad-hoc networks. MJTAR operates as a geographic routing protocol, leveraging 2-hop junction-based vehicle traffic density and curve metric distance to the destination. The protocol innovates by proposing an E-IFTIS mechanism that estimates vehicle traffic conditions up to 2-hop junctions without dependence on fixed infrastructure. Additionally, it introduces the OMSS mechanism, employing an ant colony algorithm. Notably, MJTAR surpasses GSR and GyTAR in network performance, offering a probability-based algorithm to explore multiple junction paths with low latency and a high probability of packet delivery success.

In future investigations, we aim to explore mechanisms for predicting vehicle traffic density using multi-junction-based machine-learning techniques. Furthermore, we intend to investigate forwarding node selection techniques that account for link quality to mitigate packet loss on roads between junctions. Finally, we plan to analyze the probability of packet collisions that may occur when multiple sources transmit packets to a destination in network simulations and study collision avoidance methods.

## Figures and Tables

**Table 1:** Features of summary for vehicle routing protocols.

Protocols	Junction Selection	Forwarding Strategy	Recovery Strategy	Digital Map	Single-Junction-Based Traffic-Aware	Multiple-Junction-Based Traffic-Aware	Environment
GPSR [9]	-	Greedy Forwarding	Right Hand Rule	Х	Х	Х	Highway
GPCR [23]	-	Greedy Forwarding	Right Hand Rule	Х	Х	Х	Highway
A-STAR [29]	Fixed	Greedy Forwarding	Recomputed Anchor Path	√	Х	Х	City
GSR [10]	Fixed	Greedy Forwarding	Carry and Forward	√	Х	Х	City
GyTAR [11]	Dynamic	Greedy Forwarding	Carry and Forward	√	√	Х	City
MJTAR	Dynamic	Greedy Forwarding	Carry and Forward	√	√	√	City

√: Supported, Х: Not supported.

**Table 2:** Notation description.

Notation	Description
$J_{i}$	Current Junction ID
$J_{j}$	1-Hop Neighbor Junction ID
$\tau_{ij}$	Amount of pheromone scattered between the current junction and 1-hop junction
$Dp_{j}$	Closeness of 1-hop junction to destination
$route\_p_{ij}$	Optimal route probability for neighbor 1-hop junction
$J_{k}$	2-Hop Neighbor Junction ID
$\tau_{jk}$	Amount of pheromone scattered between the current junction and 2-hop junction
$Dp_{k}$	2-hop junction to destination closeness
$route\_p_{ij}$	Optimal route probability for neighbor 2-hop junction
$route\_p_{jk}^{group\_sum}$	Group and sum the probabilities of 2-hop junctions that are in a neighbor relationship with 1-hop junctions
$d_{j}\left(x,y\right)$	Curve metric distance to destination
$D_{i}$	Curve metric Distance from the current junction to the destination
$D_{j}$	Curve metric Distance from 1-hop junction to destination
$D_{k}$	Curve metric Distance from 2-hop junction to destination

**Table 3:** Procedure for updating the CPP management table.

CPP Management Table									
One-Hop Junction List					Two-Hop Junction List				
Current Junction ID $(J_{i}$)	One-Hop Junction ID $(J_{j}$)	Amount of Pheromones $(\tau_{ij}$)	Proximity to Destination $(Dp_{j}$)	One-Hop Route Probability $(route\_p_{ij}$)	Two-Hop Junction ID $(J_{k}$)	Amount of Pheromones $(\tau_{ik}$)	Proximityto Destination $(Dp_{k}$)	Two-Hop Route Probability $(route\_p_{ik}$)	Sum the Probabilities of Same Neighbors $(route\_p_{jk}^{group\_sum}$)
J3	J2	0.13	1.33	-	J9	0.3333	1.00	-	-
J3	J4	0.5000	0.67	-	J5	0.5000	1.00	-	-
					J7	0.6667	0.33	-	
J3	J8	0.5833	0.67	-	J7	0.0000	0.33	-	-
					J9	0.5833	1	-	
					J13	0.1667	0.33	-	

**Table 4:** Procedure for calculating junction selection probabilities.

CPP Management Table									
One-Hop Junction List					Two-Hop Junction List				
Current Junction ID $(J_{i}$)	One-Hop Junction ID $(J_{j}$)	Amount of Pheromones $(\tau_{ij}$)	Proximity to Destination $(Dp_{j}$)	One-Hop Route Probability $(route\_p_{ij}$)	Two-Hop Junction ID $(J_{k}$)	Amount of Pheromones $(\tau_{ik}$)	Proximity to Destination $(Dp_{k}$)	Two-Hop Route Probability $(route\_p_{ik}$)	Sum the Probabilities of Same Neighbors $(route\_p_{jk}^{group\_sum}$)
J3	J2	0.3333	1.33	0.1333	J9	0.3333	1.00	0.0851	0.0851
J3	J4	0.5000	0.67	0.4000	J5	0.5000	1.00	0.1277	0.6383
					J7	0.6667	0.33	0.5106	
J3	J8	0.5833	0.67	0.4667	J7	0.0000	0.33	0.0000	0.2766
					J9	0.5833	1	0.1489	
					J13	0.1667	0.33	0.1277	

**Table 5:** ACO metrics creation procedure.

Ant Metrics					
Current Junction ID $(J_{i}$)	One-Hop Junction ID $(J_{j}$)	Two-Hop Junction ID $(J_{k}$)	One-Hop Route Probability $(route\_p_{ij}$)	Sum the Probabilities of Same Neighbors $(route\_p_{jk}^{group\_sum}$)	$p\_avg_{best}$
J3	J2	J9	0.1333	0.0851	0.1092
J3	J4	J7	0.4000	0.6383	0.5191
J3	J8	J9	0.4667	0.2766	0.3716

**Table 6:** Simulation setup.

SIMULATION/SCENARIO		MAC/ROUTING	
Simulator	NS-3.23	MAC	IEEE 802.11p
Simulator Time	500 s	Channel Capacity	6 Mbps
Map Size	3000 × 2400 $m^{2}$	Transmission range	170 m
Mobility model	SUM 0.32	Traffic Model	CBR
Number of vehicles	100–400	Packet size	512 bytes
Vehicle speed	20–60 km/h	Packet interval/s	0.1–1 s
Weighting factors	α = 0.5, β = 0.5	Hello interval/s	1 s

## Data Availability

The data presented in this study are available on request from the corresponding author.
